# *In-silico* drug design for the novel Karachi-NF001 strain of brain-eating amoeba: *Naegleria fowleri*

**DOI:** 10.3389/fmolb.2023.1098217

**Published:** 2023-02-09

**Authors:** Tayyab Saleem, Syed Babar Jamal, Badr Alzahrani, Amina Basheer, Sumra Wajid Abbasi, Mahwish Ali, Ashfaq Ur Rehman, Muhammad Faheem

**Affiliations:** ^1^ Department of Life Technologies, Faculty of Technology, University of Turku, Turku, Finland; ^2^ Department of Biological Sciences, National University of Medical Sciences, Rawalpindi, Pakistan; ^3^ Department of Clinical Laboratory Sciences, College of Applied Medical Sciences, Jouf University, Sakaka, Saudi Arabia; ^4^ Department of Biochemistry and Molecular Biology, University of California, Irvine, Irvine, CA, United States

**Keywords:** *Naegleria fowleri*, Karachi, Pakistan, brain-eating amoeba, pathogenic proteins, molecular docking, *in-silico*

## Abstract

*Naegleria fowleri* (*N. fowleri*) is a free-living thermophilic amoeba of fresh water and soil. The amoeba primarily feeds on bacteria but can be transmitted to humans upon contact with freshwater sources. Furthermore, this brain-eating amoeba enters the human body through the nose and travels to the brain to cause primary amebic meningoencephalitis (PAM). *N. fowleri* has been reported globally since its discovery in 1961. Recently a new strain of *N. fowleri* named Karachi-NF001 was found in a patient who had traveled from Riyadh, Saudi Arabia to Karachi in 2019. There were 15 unique genes identified in the genome of the Karachi-NF001 strain compared to all the previously reported strains of *N. fowleri* worldwide. Six of these genes encode well-known proteins. In this study, we performed *in-silico* analysis on 5 of these 6 proteins, namely, Rab family small GTPase, NADH dehydrogenase subunit 11, two Glutamine-rich protein 2 proteins (locus tags: 12086 and 12110), and Tigger transposable element-derived protein 1. We conducted homology modeling of these 5 proteins followed by their active site identification. These proteins were subjected to molecular docking against 105 anti-bacterial ligand compounds as potential drugs. Subsequently, the 10 best-docked compounds were determined for each protein and ranked according to the number of interactions and their binding energies. The highest binding energy was recorded for the two Glutamine-rich protein 2 proteins with different locus tags, and results have shown that the protein-inhibitor complex was stable throughout the simulation run. Moreover, future *in-vitro* studies could validate the findings of our *in-silico* analysis and identify potential therapeutic drugs against *N. fowleri* infections.

## 1 Introduction

*Naegleria fowleri* is a thermophilic amoeba that is free-living in nature. This single-celled microbe has a global presence, with a particular affinity for inhabiting soil and warm freshwater sources, for example, hot springs, rivers, and lakes (Marciano-Cabral and Cabral, 2007; Visvesvara et al., 2007; Visvesvara, 2010; Ali et al., 2020). In the genus of *Naegleria*, only the species of *N. fowleri* infects humans (Marciano-Cabral and Cabral, 2007; Yoder et al., 2010). In a freshwater habitat the amoeba mainly preys on bacteria to survive but occasionally finds its way into humans through the nose when they come into contact with *N. fowleri*-infested waters (Visvesvara et al., 2007; Yoder et al., 2010). Furthermore, an infection in humans manifests when *N. fowleri* makes its way to the brain causing primary amoebic meningoencephalitis (PAM), which often leads to fatalities (Yoder et al., 2010).

In terms of life cycle, *N. fowleri* exists in 3 stages. Stage 1 involves cyst formation, whereas in stages 2 and 3 the amoeba takes the form of a trophozoite and a flagellate, respectively. However, only the trophozoite state causes infection (Marciano-Cabral, 1988; Visvesvara et al., 2007). The trophozoite form is single-nucleated granular-shaped with a length of 10–35 µm. The mode of replication used by the trophozoites is binary fission, leaving the nuclear membrane unscathed in the process. Under extreme environmental conditions, like lack of nutrients, trophozoites can temporarily morph into flagellates (non-feeding in nature, with a length of 10–16 µm). Once food supply is restored, trophozoites can assume their former state. Diagnosis of trophozoites can be done using cerebrospinal tissue and fluid, whereas flagellates can only be discovered, though rarely, in the cerebrospinal fluid (CSF). However, the cyst form of the parasite is not usually detected in brain tissue. Furthermore, if the scarcity of food or coldness of temperature is prolonged, negatively impacting the growth and survival of the parasite, the trophozoite and flagellate forms turn into a cyst. The cyst state of *N. fowleri* has a diameter of 7–15 µm with a spherical shape, a single nucleus, and a smooth wall comprised of a single layer. The cyst stage is capable of resisting harsh environmental conditions, assisting the parasite to survive before the optimal conditions return (Marciano-Cabral, 1988; Visvesvara et al., 2007).

The pathogenesis of *N. fowleri* infections first involves attachment of the trophozoite form to the nasal mucosa upon entry of pathogen-containing water into the nasal cavity. Then, the pathogen uses the olfactory nerve to travel through the cribriform plate to the olfactory bulbs present in the CNS. This triggers the innate immune system and brings about a strong immune response. The trophozoite form of *N. fowleri*, which causes infection in humans, has structures called food cups on its surface. Food cups facilitate the ingestion of fungi, bacteria, and human tissue by the trophozoite. Alongside food cups, *N. fowleri* releases cytolytic molecules to accomplish the destruction of host cell and nerves. These molecules include phospholipases, acid hydrolases, phospholipolytic enzymes and neuraminidases. This pathogenicity caused by *N. fowleri* in combination with a strong immune response severely damages CNS tissues and, in most cases, eventually leads to death (Grace et al., 2015).

In 1965, Australia reported the first cases of PAM in the world (Fowler and Carter, 1965). The species of *N. fowleri* itself was first identified in 1961 after it led to infection of a fatal nature (Fowler and Carter, 1965). Shortly after, in 1962 the United States reported its first cases of *N*. *fowleri* infections in the state of Florida (Yoder et al., 2010). However, upon further inspection into the matter of *N. fowleri* infections, it was discovered that patients from Virginia may have suffered from PAM as far back as 1937, based on the analysis of autopsy tissue samples retrieved from the archives (Dos Santos, 1970).

With data from around the world concerning *N. fowleri* infections in humans being sparse, given the infrequency of infections, Karachi (the largest city in Pakistan) in recent years has emerged as a major hunting ground for this brain-eating amoeba. In 2008, the first PAM case emerged in Karachi (Ali et al., 2020). By 2019 the number had reached 146 in just over a decade, which superseded the total number of cases reported in the United States between 1968 and 2019 (Ali et al., 2020). A major cause for concern. Furthermore, in Karachi *N. fowleri* is reported to mainly spread through water, causing PAM, making it a waterborne pathogen. Waterborne diseases make up 80% of all the diseases present in Pakistan, leading to 33% of all deaths in the country. These water-borne diseases can include cancer, caused by factors such as the release of industrial waste and pesticides into ground and surface water. Furthermore, Pakistan annually experiences 2.5 million diarrhea-related deaths caused by the contamination of groundwater with bacterial, viral, and protozoal pathogens. Examples of these pathogens include *Enterobacter*, *E. coli*, *Salmonella*, *Giardia lamblia*, *Clostridium,* and *Cyclosporin coetaneities*. Among other waterborne diseases in Pakistan, hepatitis E is reported to have the highest prevalence in the capital city of Pakistan, Islamabad, because of the untreated water supply (Qamar et al., 2022).

Furthermore, most PAM cases reported in the United States involved children aged less than 14 (Ali et al., 2020), whereas in Pakistan most of the infected patients were adults between 26 and 45 years of age (Naqvi et al., 2016; Ali et al., 2020). This suggested the possibility of a genetic variation between the Karachi strain of *N. fowleri* compared to previously reported strains around the world. This suggestion was recently validated by whole genome sequencing of the Karachi strain (Karachi-NF001), isolated from one of the reported cases (male patient, 59 years of age) who had traveled from Riyadh, Saudi Arabia in June of 2019 to Karachi (Guan et al., 2021). The sequencing yielded 15 unique genes, 6 of them with known protein products, not found in the genome of any other reported *N. fowleri* strains worldwide (Guan et al., 2021).

In terms of treatment, since the occurrence of PAM in humans is rare globally, no standardized treatment strategies for *N. fowleri* exist in the literature. Furthermore, no clinical trials have been conducted to test possible drug candidates against this life-threatening parasite (Grace et al., 2015). So far, only *in-vitro* studies and case reports have contributed in terms of identifying possible medications for PAM cases, some of which are currently in use. These medications include amphotericin B, fluconazole, miltefosine, miconazole, rifampin, and azithromycin (Grace et al., 2015). However, to our knowledge, there are no published studies proposing any drug compounds for use against this novel strain of *N. fowleri* from Karachi. Therefore, the purpose of the present *in-silico* study is to analyze the known protein products of 5 of the 15 unique genes, identified in the Karachi-NF001 strain (Guan et al., 2021), by determining their structure, active sites, and docking them with selected ligand compounds (potential drug candidates) to treat the novel Karachi-NF001 strain.

## 2 Materials and methods

### 2.1 Retrieval of protein sequences

**FIGURE 1:**
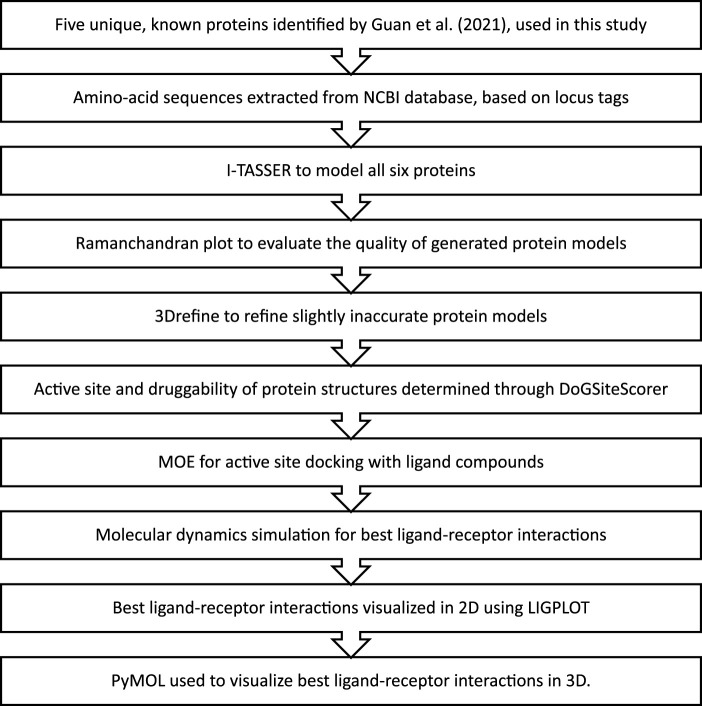
The methodology followed for *in-silico* drug design of the Karachi-NF001 strain of *Naegleria fowleri*.

Out of the 15 unique genes discovered by Guan et al. (2021) in the Karachi-NF001 strain, only 5 with known protein products were used in this study. These proteins were Rab family small GTPase, NADH dehydrogenase subunit 11, two Glutamine-rich protein 2 proteins (locus tags: 12086 and 12110), and Tigger transposable element-derived protein 1. The respective amino acid sequences for these proteins were obtained according to the method described by Qureshi et al. (2021), using locus tags: 2204, 11435, 12086, 12110, and 12116, provided in the literature (Guan et al., 2021). The complete workflow of this study is illustrated in Figure 1.

### 2.2 Protein sequence and structure determination

After sequence retrieval, the I-TASSER tool (Sehgal, 2017), a protein modeling software, was used to create the respective models for all six proteins (Yang et al., 2015; Yang and Zhang, 2015; Zheng et al., 2021). For each protein, I-TASSER software generated multiple protein models. The model with the highest C-score was selected for each protein. C-score represents the confidence score which is dependent on the matching of the fold of the provided protein template with other known proteins with which they do not share homology, and a structure is determined. Therefore, a higher C-score signifies a better fold recognition of the provided template (Roy et al., 2010).

These structures, in PDB format, were inserted into the Ramachandran plot online tool (Anderson et al., 2005a). The Ramachandran plot uses dihedral angles or torsion angles to present all possible structures of the protein in a two-dimensional graph. After that, the software generates three types of observations to signify the quality of its predictions (1. Highly preferred, 2. Preferred, and 3. Questionable). Only the highly preferred observations, for the obtained protein models, which showed a percentage of more than 88% were directly used in further steps of active site determination. The protein structures with a percentage of less than 88% were refined with the 3Drefine online tool. The tool itself relies on optimizing the hydrogen bonding and minimizing the energy of atoms in the protein structure. Furthermore, during the refinement of a protein structure, the software attempts to convert slightly inaccurate protein models to their respective native states through adequate folding and assemblage which allows the structure of the protein to exist in an operative and functional form. Following the use of 3Drefine the refined structures of the proteins were once again inserted into the Ramachandran plot to confirm the percentage to be above 88% (Bhattacharya and Cheng, 2013a; Bhattacharya and Cheng, 2013b; Bhattacharya et al., 2016).

### 2.3 Active-site identification

Catalytic pockets and their druggability, for each of the 5 known proteins, were identified using the DoGSiteScorer online tool (Volkamer et al., 2010; Volkamer et al., 2012). The tool uses PDB files and generates catalytic pocket-containing structures. It then provides a “drug score” to assist in measuring the druggability of the inserted proteins, this score lies between 0 and 1. A usable drug score is usually more than 0.6, whereas a favorable score usually supersedes 0.8.

### 2.4 Molecular docking of potential proteins with the ligands

Protein-ligand docking aims to accomplish the objective of accurately predicting the location and orientation of a ligand within the binding pocket of a particular receptor. The docking of proteins to their ligands has seen widespread application in modern drug development. To determine the ligand interaction with receptor and to visualize docking of ligand compounds against protein active sites, the Molecular Operating Environment (MOE) software was used (Tomar et al., 2010). MOE is designed by the Chemical Computing Group to support Cheminformatics, Molecular Modeling, Bioinformatics, Virtual Screening, Structure-based-drug-design and can be used to build new applications based on SVL (Scientific Vector Language). The following infrastructure: Intel (R) xenon (R) CPU E5620@2.40GHz system having 3.8 GB RAM with the open 11.4 (X 86_64) operating platform was used.

For docking, 105 ligands were retrieved from the literature (Morris, 2003; Park et al., 2006; Shan et al., 2008; Ibis et al., 2013), which had been used in previous studies as antimicrobial compounds but were never used against *N. fowleri*. After that, docking, using MOE with default parameters, was performed involving proteins and ligand compounds that were prepared and minimized. The software generated a docking score (S) and the number of interactions for each ligand was used to select the best compounds with the highest S score and interactions. These ligands were then analyzed using PyMOL an open-source molecular visualization system (Sehgal et al., 2014; Yuan et al., 2017).

### 2.5 Validation and visualization of interacting residues

The docking results obtained *via* MOE were validated by calculating the RMSD values, followed by further analysis using Ligplot (Sehgal et al., 2015; Sehgal et al., 2016). The LIGPLOT software automatically creates schematic 2-D representations of protein-ligand interactions from standard Protein Data Bank (PDB) file input (Wallace et al., 1995; Laskowski and Swindells, 2011), and PyMOL which is capable of creating videos and images of excellent quality that depict macromolecules in a variety of diverse 3D representations, such as ribbons, dots, cartoons, surfaces, sticks, and lines.

### 2.6 MD simulation analysis

To verify the docking results one of the proteins which had highest binding affinity with the inhibitor was further validated by Molecular dynamic simulations by AMBER16 (Case et al., 2014). The best protein docked solution and best-characterized inhibitor were put through a 50-ns MD simulation production run. An AMBER, (GAFF) force field (Wang et al., 2004) was used to optimize the inhibitor, whereas the ff14SB force field (Case et al., 2016) was used to produce the protein parameters. Padding distance was adjusted at 12 between protein and box borders in order to achieve complex integration into a TIP3P water box. By adding Na + ions to the system, it was neutralized following that, in langevin dynamics, the system was heated to 300 K (NVT) for 20 ps to keep the temperature constant (Lavenda, 2016). At a time, step of 2-fs, a 5 kcal/mol-A2 restriction on carbon alpha atoms was permitted. System was relaxed for 100 milliseconds during equilibration. A 50-ps NPT ensemble was used to maintain system pressure. Finally, at a rate of 2-fs, a 50-ns manufacturing run was completed. The AMBER CPPTRAJ program was used to examine the generated trajectories for structural parameters. The hydrogen bonds formed between best docked protein and the inhibitor throughout the trajectories were displayed in VMD with a 30° angle and a 0.35 nm bond distance (Roe and Cheatham, 2013).

## 3 Results and discussion

Amongst the 15 unique genes identified in the Karachi-NF001 strain of *N. fowleri* by Guan et al. (2021), only 6 had known protein products and only 5 of them were the focus of this study (Table 1). These proteins were: Rab family small GTPase, NADH dehydrogenase subunit 11, Glutamine-rich protein 2 (locus tags: 12086 and 12110) and Tigger transposable element-derived protein 1. Furthermore, after retrieving the protein sequences for all 5 proteins (Qureshi et al., 2021), they were modelled using the I-TASSER software (Yang et al., 2015; Yang and Zhang, 2015), and their catalytic pockets were determined using the DogSiteScorer software (Volkamer et al., 2010; Volkamer et al., 2012). For ligand docking, a total of 105 antibacterial compounds were extracted from published literature (Morris, 2003; Park et al., 2006; Shan et al., 2008; Ibis et al., 2013), and used as ligand compounds in the MOE docking software (Tomar et al., 2010). The ligand compounds were then docked against all six proteins separately and ranked based on their binding affinities for the respective proteins. Furthermore, for each protein, only the ligand compound with the highest binding energy was selected for molecular dynamics simulation and its interaction was visualized using the PyMOL software (Yuan et al., 2017).

**TABLE 1:** The MOE docking score of drug targets with their ligands. The highest binding energies are shown by a bold black color.

Protein locus tag	Protein name	Name of the ligand	Number of interactions	Residues	Docking score (S)	MM/GBVI
2204	Rab family small GTPase	6,7-dichloro-5,8-dimethoxynaphthalene-1,4-dione	2	Ser 145	−13.0532	−14.769
				Ser 149		
11435	NADH dehydrogenase subunit 11	5-hydroxy-2-methylnaphthalene-1,4-dione	4	His 176	−12.9604	−13.599
				Ser 381		
				Ser 381		
				Asp 424		
12086	Glutamine-rich protein 2	5,8-dihydroxy-2,3-dihydronaphthalene-1,4-dione	6	Gly 303	−11.7454	−**18.147**
				Asn 305		
				Glu 257		
				Gln 214		
				Gln 214		
				Arg 306		
12110	Glutamine-rich protein 2	1,4,5,8-tetramethoxynaphthalene	9	Arg 169	−16.9696	**−20.89**
				Arg 169		
				Asp 122		
				Asp 122		
				Gln 113		
				His 172		
				Ser 174		
				Thr 173		
				Thr 173		
12116	Tigger transposable element-derived protein 1	1,4,5,8-tetramethoxynaphthalene	3	Arg 182	−10.995	−14.96
				Asn 175		
				Asn 175		

### 3.1 Rab family small GTPase

#### 3.1.1 Description

Rab family small GTPase protein is encoded by the Rab1b gene in humans and has a molecular weight of 22.171 kDa. It is important in regulating membrane trafficking inside the cell, from formation to membrane fusion of transport vesicles. Rab proteins have inactive (bound by GDP) and active (bound by GTP) forms which they switch between, which assists in the recruitment of downstream effectors of different kinds to membranes that play an important role not only in the formation and fusion of vesicles but also in their movement and tethering. Furthermore, Rabs are involved in the early development of the autophagic vacuole in the endoplasmic reticulum (Homma et al., 2021).

#### 3.1.2 Docking results

**FIGURE 2:**
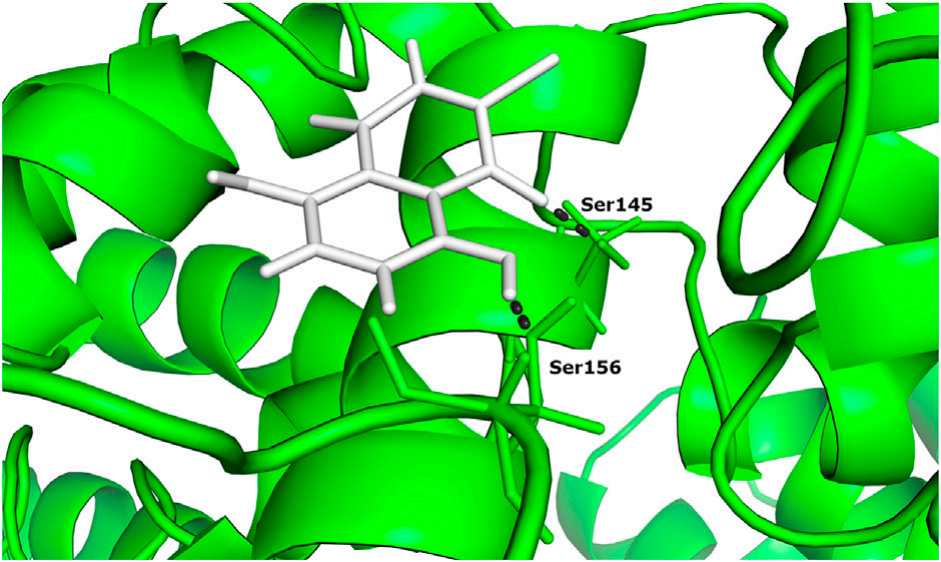
The docked complex of 6,7-dichloro-5,8-dimethoxynaphthalene-1,4-dione and Rab family small GTPase protein.

One of the 105 ligand compounds used in this study, 6,7-dichloro-5,8 dimethoxynaphthalene-1,4-dione showed the highest binding affinity for Rab family small GTPase with the binding energy value of −14.769 Kcal/mol. Furthermore, 2 of its interacting residues that are involved in protein interaction are Ser145 and Ser 149. The interaction can be seen in more detail in Figure 2.

### 3.2 NADH dehydrogenase subunit 11

#### 3.2.1 Description

NADH dehydrogenase subunit 11 is an accessory subunit of the Complex I of NADH dehydrogenase that is present in the respiratory chain of the mitochondrial membrane, and it does not participate in catalysis. It is encoded by the NDUFB11 gene in humans. Electrons from NADH are transferred to the respiratory chain with the help of Complex I, and ubiquinone acts as the immediate acceptor of electrons in the enzyme. The molecular weight of NADH dehydrogenase subunit 11 protein is 17.317 kDa (Čermáková et al., 2021).

#### 3.2.2 Docking results

**FIGURE 3:**
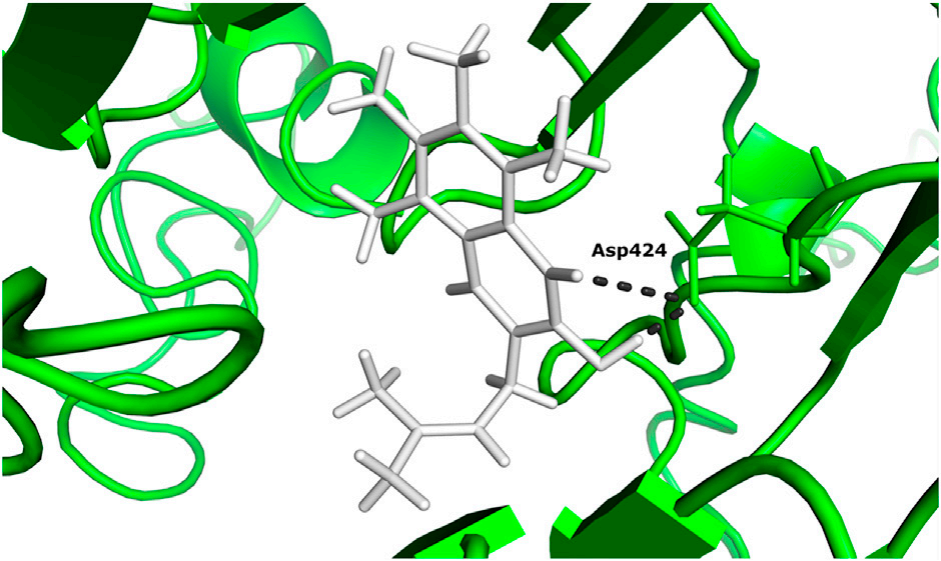
The docked complex of 5-hydroxy-2-methylnaphthalene-1,4-dione compound with NADH dehydrogenase subunit 11 protein.

According to the docking results (Table 1), 5-hydroxy-2-methylnaphthalene-1,4-dione interacts with NADH dehydrogenase subunit 11 protein with the highest binding energy of -13.599 Kcal/mol. Furthermore, the interaction between the residue Asp 424 and NADH dehydrogenase subunit 11 is shown in Figure 3.

### 3.3 Glutamine-rich protein 2

#### 3.3.1 Description

Glutamine-rich protein 2, encoded by the QRICH2 gene in humans (Hiltpold et al., 2022), has a molecular weight of 180.827 kDa and plays an important role in cell projection organization, sperm flagella synthesis, and maintenance of the flagellar structure. Also, it suppresses the degradation and ubiquitination of proteins that function in the development and motility of flagella (Kherraf et al., 2019).

#### 3.3.2 Docking results

**FIGURE 4:**
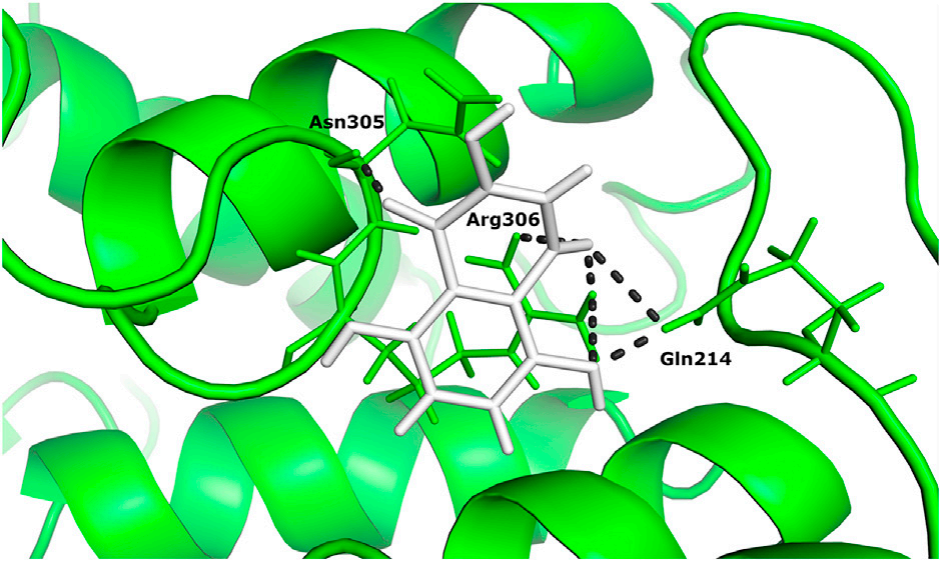
The docked complex of 5,8-dihydroxy-2,3-dihydronaphthalene- 1,4-dione with Glutamine-rich protein 2 at locus tag 12086.

**FIGURE 5:**
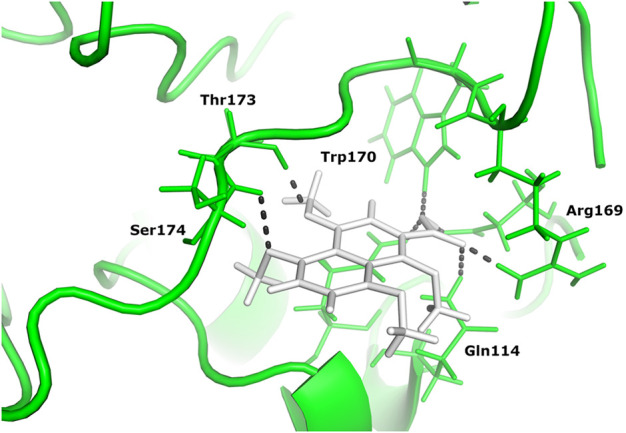
The docked complex of 1,4,5,8-tetramethoxynaphthalene and Glutamine-rich protein 2 at locus tag 12110.

Two different ligand compounds 5,8-dihydroxy-2,3-dihydronaphthalene-1,4-dione and 1,4,5,8-tetramethoxynaphthalene show maximum binding affinity for the two Glutamine-rich protein 2 proteins encoded by different genes (Locus tags: 12086 and 12110). Their respective binding energies are −18.147 Kcal/mol and −20.890 Kcal/mol (Table 1). Furthermore, the interacting residues for 5,8-dihydroxy-2,3-dihydronaphthalene-1,4-dione are 6 in total and include Gly 303, Asn 305, Glu 257, Gln 214, Gln 214, and Arg 306 (Figure 4). However, 1,4,5,8-tetramethoxynaphthalene interacts with 9 residues Arg 169, Arg 169, Asp 122, Asp 122, Gln 113, His 172, Ser 174, Thr 173 and Thr 173 (Figure 5).

### 3.4 Tigger transposable element-derived protein 1

#### 3.4.1 Description

Tigger transposable element-derived protein 1 is encoded by the TIGD1 gene in humans and has a molecular weight of 67.299 kDa. In humans, it is part of the tigger subfamily which belongs to the superfamily of pogo DNA transposons. Furthermore, TIGD1 shares a relationship with fungal and Nematodal DNA-mediated transposons but is only vaguely related to the mariner and Tc1 transposases. Also, there is a strong similarity between TIGD1 and CENP-B (Mammalian Centromere Protein B). Still, the primary function of the TIGD1 gene is unknown (Cam et al., 2008).

#### 3.4.2 Docking results

**FIGURE 6:**
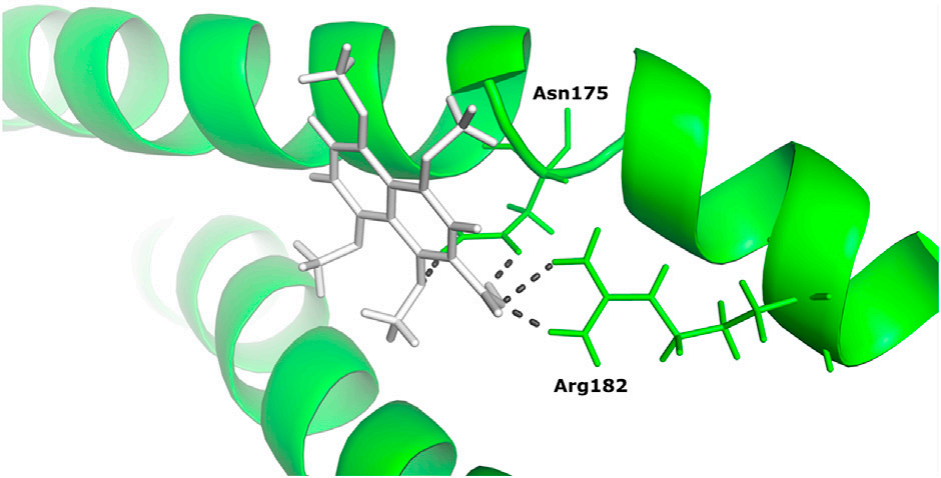
The docked complex of 1,4,5,8-tetramethoxynaphthalene with Tigger transposable element-derived protein 1.

Based on binding affinity, 1,4,5,8-tetramethoxynaphthalene interacts with Tigger transposable element-derived protein 1 with the highest binding energy of any other ligand compound used in this study. With a value of −14.960 Kcal/mol. The interacting residues, include Arg 182, Asn 175. (Figure 6).

Table 1 shows the ligands which showed the highest binding affinity for each of these 5 respective proteins, along with their name and the interacting residues and number of interactions they share with each protein. With the use of the molecular mechanics/generalized Born volume integration (MM/GBVI) model, we calculated the binding free energy of each compound ligand.

### 3.5 MD simulation of highest binding affinity protein (glutamine-rich protein 2)

**FIGURE 7:**
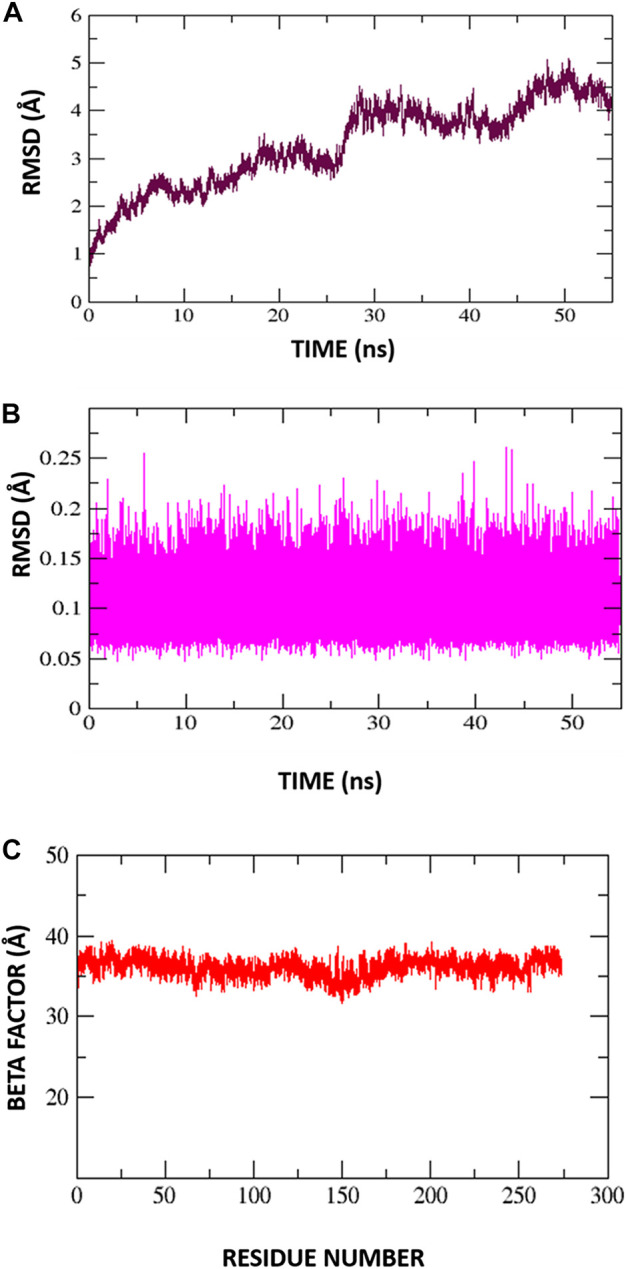
Molecular dynamic simulation exploration for Protein-Inhibitor complex. **(A)** C-RMSD, **(B)** L-RMSD, **(C)** Beta factor.

Root mean square deviations of the complex (C-RMSD), root mean square deviations of the ligand (L-RMSD), and beta factor (-factor) were used to assess the output trajectory pattern. In Figures 7A–C all three parameters are shown. To interpret structural differences among the stacked pictures acquired by MD simulation, RMSD was computed for the protein Carbon atoms. The system’s mean RMSD for the protein is 3.28474 (with a maximum of 5.098 measured at frame 50360). Generally, the RMSD plot is suggesting the structure has undergone conformational variations followed by a stable trend towards the end. To understand these minor variations, snapshots at 0-ns, 10.76-ns, 20.8-ns, 36.2-ns, and 44.2-ns were extracted from the trajectories and analyzed. No global conformational changes were seen though quite frequently local secondary structure interconversion were revealed at different intervals. One possible reason for such local structure changes may be due to little adjustments acquired by the inhibitor during simulation. Following they, to confirm the ligand binding stability with the receptors, the ligand RMSD was performed after the receptor RMSD (Figure 7B). For the ligand molecule, an average RMSD of 0.105462 Å and a maximum of 0.2608 Å at 43 ns were reported. However, these very minor alterations are in favor of the higher system stability. Again, small fluctuations in ligand RMSD were observed, representing conformational shifts as previously indicated. Furthermore, the β-factor (Figure 7C) reflected high thermal stability of the system with average score of 36.1278 Å^2^ with highest of 39.3162 Å^2^. This illustrates the protein residues with very stable behavior following the inhibitor engagement.

## 4 Conclusion

The recently discovered strain of *N. fowleri* in Karachi, Karachi-NF001, is a lethal strain that requires a treatment strategy. Therefore, in this *in-silico* study we docked the known protein products of 5 unique genes identified in the genome of Karachi-NF001 against 105 selected antibacterial compounds from the literature. The ligand compounds were ranked based on their binding affinity for these 5 proteins. Based on our findings, the ligand compounds showed the highest binding affinity for Glutamine-rich protein 2, from two different locus tags (12086 and 12110), and therefore should be considered as potential drug targets. MD simulation of protein with the inhibitor showed that protein inhibitor complex was stable. Furthermore, *in-vitro* studies can provide significant insight to validate the findings of our *in-silico* analysis followed by possible *in-vivo* studies of these compounds as alternative therapeutics against *N. fowleri*.

## Data Availability

The datasets presented in this study can be found in online repositories. The names of the repository/repositories and accession number(s) can be found below: https://www.ncbi.nlm.nih.gov/, 2204, 11435, 12086, 12110, 12116.
